# Compartmentalized evolution of Bovine Viral Diarrhoea Virus type 2 in an immunotolerant persistently infected cow

**DOI:** 10.1038/s41598-019-52023-w

**Published:** 2019-10-29

**Authors:** Barbara Colitti, Chiara Nogarol, Mario Giacobini, Maria Teresa Capucchio, Ilaria Biasato, Sergio Rosati, Luigi Bertolotti

**Affiliations:** 1 Department of Veterinary Science, University of Torino, Largo Paolo Braccini 2, 10095 Grugliasco, Torino Italy; 2 Department of Agricultural, Forestry and Food Sciences, University of Torino, Largo Paolo Braccini 2, 10095 Grugliasco, Torino Italy

**Keywords:** Molecular evolution, Bioinformatics, Viral evolution

## Abstract

Bovine viral diarrhea virus (BVDV) is one of the most important pathogens of cattle worldwide. BVDV-1 is widely distributed in Italy, while BVDV-2 has been detected occasionally. BVDV can be classified in two biotypes, cytopathic (CP) or noncytopathic (NCP). The characteristic of the virus is linked with the infection of a pregnant dam with a NCP strain: due to viral establishment before maturation of the fetal immune system the calf remains persistently infected (PI) and immunotolerant to the infecting BVDV strain. Thanks to their immunotolerance, PI animals represent a unique model to study the viral distribution and compartmentalization in absence of immunoresponse *in vivo*. In the present study, NGS sequencing was used to characterize the BVDV2 viral strain infecting a PI calf and to describe the viral quasispecies in tissues. Even if the consensus sequences obtained by all the samples were highly similar, quasispecies was described evaluating the presence and the frequency of variants among all the sequencing reads in each tissue. The results suggest a high heterogeneity of the infecting viral strain suggesting viral compartmentalization. The quasispecies analysis highlights the complex dynamics of viral population structure and can increase the knowledge about viral evolution in BVDV-2 persistently infected animals.

## Introduction

Bovine viral diarrhea virus (BVDV) is a member of the genus *Pestivirus*, family Flaviviridae. BVDV is a single-stranded, positive sense RNA virus of approximately 12.5 kb in size^1^ and it can be divided into 2 species (1 and 2) and several subtypes. BVDV-1 includes the classical European isolates, commonly used in laboratory diagnosis and vaccine production. BVDV-2 was initially identified in severe outbreaks of acute infection in North America^2^ but low virulence strains were reported also in Europe^3^ and occasionally in Italy^4,5^. Rare is the identification of Hobi-like virus, tentatively classified as possible third species of BVDV^6–8^.

BVDV can also be categorized in two biotypes, cytopathic (CP) and non-cytopathic (NCP), based on its ability to lyse cells in tissue cultures and its role in the fatal Mucosal Disease (MD) in persistently infected (PI) animals^9^. The MD is caused by the mutation of the NCP to CP biotype, often caused by massive changes in the viral genome (i.e. recombination between viral and host genome in the viral NS2-NS3-NS4 genome region)^9–11^. Infections with NCP strains during the first half of pregnancy may lead to persistent infection of the calf. Since the infection occurs before the maturation of the fetal immune-system, viral proteins are considered as self antigens and the PI calf remains immunotolerant to the BVDV strain, allowing the viral replication in all body compartments and the viral shedding during the entire animal life. For this reason, PI animals are considered the most important viral reservoir within the herd, as well as the cows harboring a PI fetus can be considered the main viral diffusion route among herds^12^.

RNA viruses can mutate rapidly due to errors induced by an error-prone RNA-dependent RNA polymerase. The high level of mutation rate can drive the virus to produce an heterogeneous population of closely related variants, known as mutant spectrum or quasispecies. This population diversity is known to be important in RNA virus evolution and survival, and many studies were conducted to explain viral quasispecies in RNA viruses^13,14^. Moreover, the importance of quasispecies during chronic or persistent infections is widely accepted for many diseases and is thought to contribute to the viral pathogenesis^15–20^. The intra-host variability represents a substrate for the selective pressure exerted by the immune system of the host which leads to the continuous evolution of viruses and its adaptability in the event of environmental changes^13^. One of the mechanisms played out by the virus in order to evade the host’s defenses is the compartmentalization of variants as demonstrated for human HCV and HIV viruses^21–26^.

Several studies have been conducted in order to investigate the quasispecies behavior in pestiviruses^27,28^. A high degree of variability is reported in the BVDV E2 membrane glycoprotein sequence^29,30^, while the NS3 and NS5 region are recognized as more conserved due to their association with vital functions^29,31,32^. The correlation between genome mutation and the appearance of the CP biotype is widely accepted^33–35^ but little is known about how this modification occurs, namely if the CP strain emergence is caused by a single mutation event or by a progressive divergence of the NCP strain.

Cloning of PCR products and Sanger sequencing have been extensively used as reference techniques to study viral populations. However, these procedures are time-consuming and expensive, with biased outputs^36,37^ making more difficult the study of viral population in considerable detail as they stop at consensus-level sequencing.

Next generation sequencing (NGS) technologies represent an opportunity to deepen the analysis of RNA virus variants^29,38,39^ by revealing nucleotide substitutions present in only a small fraction of the population.

Moreover, the lack of immune response in BVDV PI animals, which allows the virus to replicate without an apparent selective pressure from the immune system, makes these subjects a unique model to study viral diversity and tissue compartmentalization *in vivo*.

The aim of this study is to investigate BVDV-2 heterogeneity in different body compartments and to describe the quasispecies diversity in a PI cattle through analysis of different regions of viral genome with a NGS approach.

## Results

**Figure 1:**
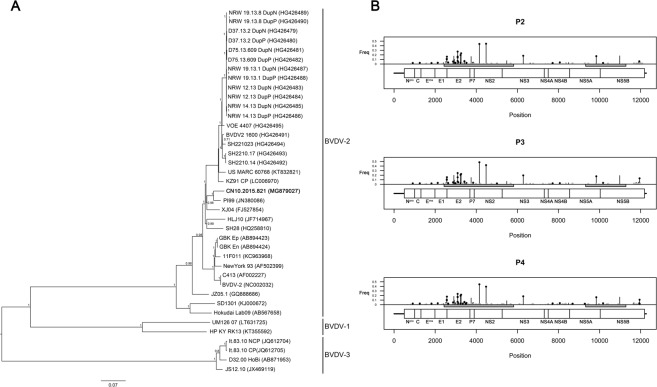
BVDV-2 full genome characterization. Panel A: Bayesian tree based on the aligned full genome sequences, including all the three BVDV viral species; GenBank accession number is reported between brackets; BVDV strain CN10.2015.821 is reported in bold; posterior probabilities of each node is reported. Panel B: variable sites are reported along viral genome; Gray rectangles below the genome structure indicated the regions amplified during amplicon sequencing procedures for tissues characterization. Sites with a frequency variation greater than 0.02 are reported in black, otherwise they are reported in gray. Non synonymous variable sites are marked with a black or gray dot at the top of bars.

The full-length genome sequence was obtained from the three passages in cell culture. No clear evidences of cell culture adaptation were noted. Indeed the consensus sequences of all the three passages were 100% identical. The sequence obtained from the second cell culture passage was aligned with reference sequences available in GenBank and Bayesian tree was built. The tree topology clearly shows that CN10.2015.821 strain belongs to the BVDV-2 clade (Fig. 1A). Identification of variable sites along the genome revealed that mutations (n = 31 with a frequency ≥ 0.02) are particularly concentrated in the first half of the genome, in the region including E2 and NS2-3 genes. Moreover, the larger part of the mutations was non synonymous (Fig. 1B).

Tissue amplicon sequencing was performed in five different Illumina MiSeq runs, with an average output of 1 million reads per tissue. No difference in terms of reads quality and quantity was observed among the five runs. Tissue amplicons analyses were conducted, and consensus sequences alignment showed a very high similarity. Along the investigated region, obtained by concatenating the consensus sequences of the three amplified genome segments, the nucleotide difference ranged between 0% and 0.25%, suggesting a very low heterogeneity among tissues and the absence of clear compartmentalization. The only relevant change along the alignment is the codon AAA insertion showed by cerebellum and cortex tissue sequences, corresponding to an extra lysine at the beginning of the E2 sequence (at position 2625 referring to CN10.2015.821 BVDV-2 strain, GenBank accession number: MG879027).

The further analyses conducted on the within-tissue sequence variation showed a more heterogeneous scenario. Variable positions (VS) were identified and were compared among all the tissues, considering a more conservative frequency threshold of 2%. Most of the VS recorded in the full genome analysis (28 out of 31) were detected in tissue homologous regions. The largest part of all the VS are in the E2 coding region. The same AAA insertion described in cerebellum and cortex samples was also recorded with a lower frequency in spinal cord sample (21%), in the optic nerve (2.5%) and in the pituitary gland (2.3%), thus supporting the different evolutionary path in the tissues belonging to the nervous system. The number of VS differed among tissues and the number of non synonymous substitutions was statistically larger than the synonymous ones (Wilcoxon signed rank test p < 0.001, Table 1). The ratio between dN (the number of non synonymous substitutions per non synonymous site) and dS (the number of synonymous substitutions per synonymous site) was calculated for each tissue (Tab. 1). The results showed diversified behaviors. Some tissues showed a dN/dS ratio greater than 1, suggesting the presence of selective pressure and tissue adaptation. As reported in Table 1, most of the samples from lymph nodes, skin and oral mucosa, spleen, kidney, pituitary gland and ileocecal valve showed ω grater than one. On the other hand the remaining tissues, including the ones belonging to the nervous system, showed ω lower than 1.

**Table 1:** List of analyzed tissues.

Id	Tissue	N	S	dN	dS	ω
1	Skin	23	7	0.0005903	0.0005125	1.15
2	Subscapular ln	34	10	0.0007163	0.0005128	1.40
6	Submandibular ln	34	9	0.0006810	0.0005023	1.36
7	Retropharyngeal ln	7	5	0.0001585	0.0002209	0.72
9	Tonsil	7	4	0.0001281	0.0003078	0.42
10	Thymus	24	12	0.0005019	0.0008652	0.58
12	Oral mucosa	17	3	0.0005856	0.0004316	1.36
16	Bronchus	18	7	0.0004887	0.0005182	0.94
17	Lung	20	5	0.0006423	0.0009176	0.70
18	Mediastinal ln	36	9	0.0007761	0.0005817	1.33
21	Spleen	30	7	0.0005700	0.0004765	1.20
23	Kidney	21	3	0.0004626	0.0004091	1.13
24	Liver	21	6	0.0006162	0.0006747	0.91
27	Ovary	13	5	0.0004416	0.0004627	0.95
29	Pituitary gland	19	6	0.0004463	0.0003657	1.22
31	Mesenteric ln	26	7	0.0007515	0.0006082	1.24
35	Ileocecal ln	35	10	0.0006242	0.0006585	0.95
36	Ileocecal valve	19	4	0.0003914	0.0003465	1.13
39	Rectum	19	3	0.0005396	0.0006903	0.78
40	Rumen	15	5	0.0003337	0.0003378	0.99
44	Optic nerve	18	13	0.0006680	0.0007293	0.92
45	Spinal cord marrow	25	13	0.0005956	0.0010582	0.56
47	Cerebellum	47	23	0.0007995	0.0011246	0.71
48	Cortex	18	15	0.0003500	0.0009980	0.35
52	Bone marrow	11	4	0.0003160	0.0006448	0.49

Number of synonymous (S) and non synonymous (N) variable sites is reported, as well as the the number of non synonymous substitutions per non synonnymous site (dN), the the number of synonymous substitutions per synonnymous site (dS) and the dN/dS ratio (ω).

**Figure 2:**
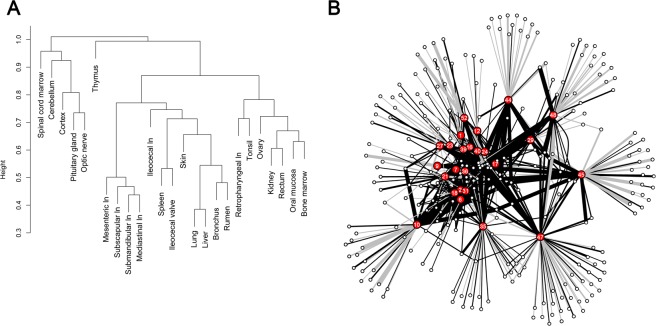
Relationship among tissues based on shared VS. Panel A: dendrogram based on Dice similarity matrix and complete linkage cluster algorithm. Panel B: bipartite network; tissue nodes are reported in red, and numbered according to Table 1; VS nodes are reported in white; synonymous VS are linked to nodes with gray edges, while the non synonymous ones are linked to nodes with black edges. Edge width is proportional to VS frequency variation.

For all the tissues the list of every possible VS was considered and codified with the presence/absence of variants. The comparison among all the patterns was performed by evaluating the Dice similarity index and the differences among tissue patterns were visualized by cluster dendrogram (Fig. 2A). The dendrogram topology clearly shows how tissues from the nervous system form a separate clade and the largest part of lymph nodes clustered together.

The same distance matrix was used to draw a bipartite network (Fig. 2B). Tissues are represented by nodes, while the VS are divided into two separated families and the edges are weighted by the frequency of each VS. Variable sites nodes showed a high heterogeneity in the degree distribution with nodes’ degrees ranging from 1 to 25. The VS nodes with degree equal to 1 (n = 158) represent the VS typical of each tissue, whereas the other 92 VS nodes show higher degree. Ten VSs showed a degree greater than 20, picturing a set of common variable positions for all the tissues.

**Figure 3:**
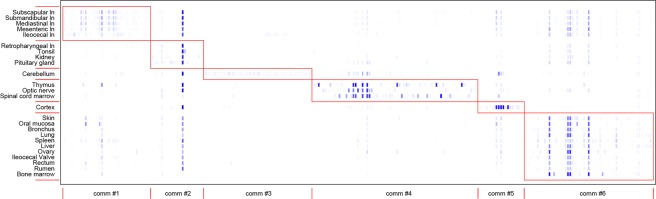
Communities detection. Modules identified by Beckett algorithm are boxed and numbered on the x axis. Tissues belonging to each module are reported in the y axis. The frequency of VS within each module is indicated by blue color depth (i.e. light blue indicates low frequency VS, dark blue indicates high frequency VS).

Beckett algorithm^40^ was applied to identify possible communities within the network, taking into account the network bipartite structure and frequency of each mutation (Fig. 3).

The algorithm highlighted the presence of 6 modules (i.e. communities). Community #1 includes only tissues from lymph nodes (subscapular, submandibular, mediastinal, mesenteric, and ileocecal lymph nodes). Nodes from the retropharyngeal lymph node, tonsils, kidney and pituitary gland belong to the Community #2. Cerebellum and cortex are identified as two different groups (communities #3 and #5), close to the community #4, which includes the other samples from the nervous system (optic nerve and spinal cord marrow) and the thymus. The largest community includes the remaining tissues.

**Figure 4:**
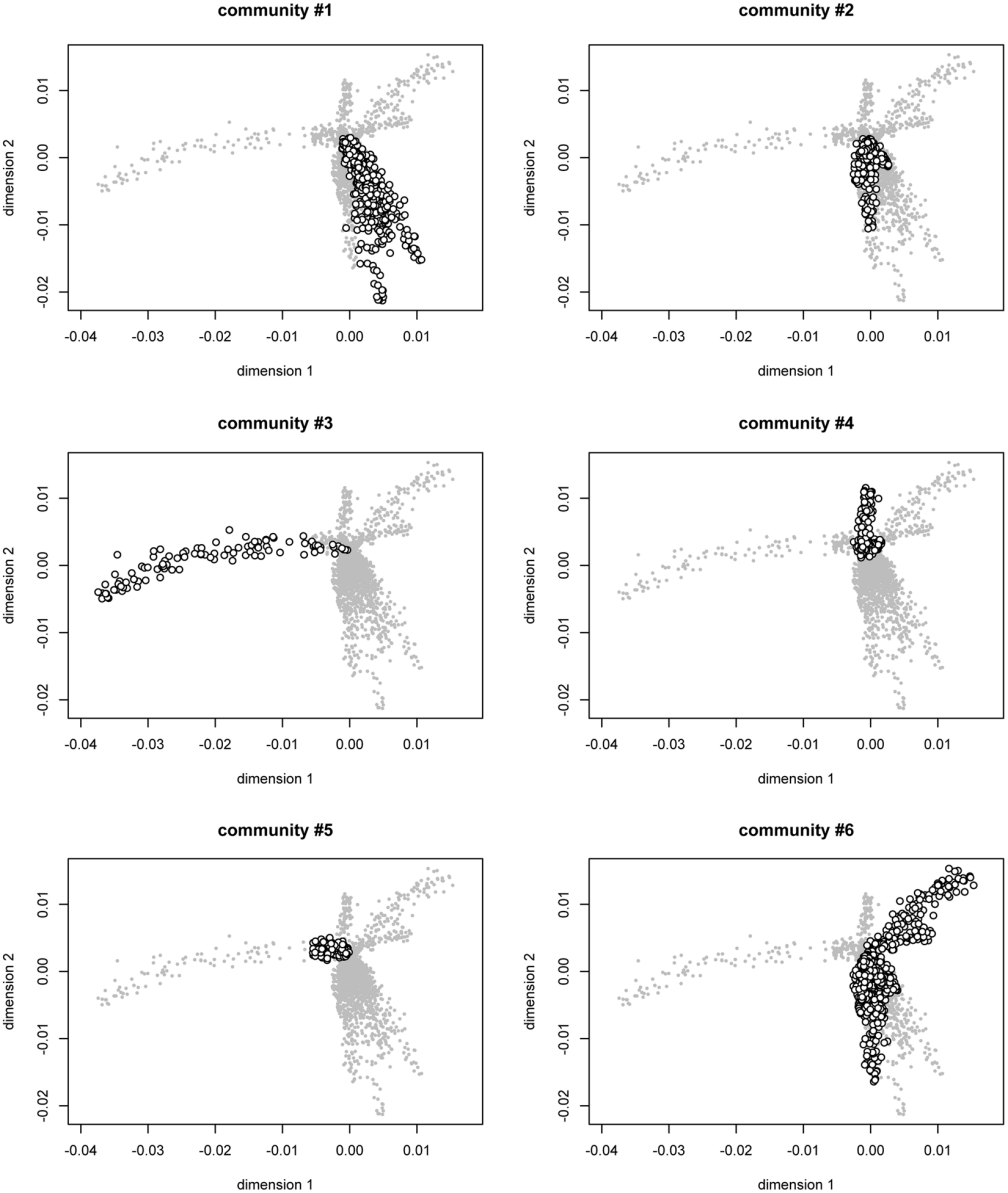
Subpopulation structure of BVDV-2. Multidimensional scaling plot based on the uncorrected p distance matrix calculated on random sequences. All sequences are represented in gray. Each community structure is reported in a different panel and sequences are indicated as white points.

Finally, random sequences based on the VS position/frequency were generated and compared. Uncorrected p-based distance matrix was used to reconstruct a two-dimensional scaling plot (Fig. 4). The results obtained by random sequences analysis described the possible structure of viral quasispecies within each tissue. The clustering detected by community algorithm is respected, suggesting different evolutionary patterns and variable divergence extent. Random sequences belonging to each tissue create a swarm around the consensus sequence, representing the heterogeneity within each compartment. However, if random sequences belonging to different tissues are compared, the differences among them contribute to understand the genetic and evolutionary relationships among the viral subpopulations. In more details, some tissue shared the same evolutionary landscapes, resulting in a stronger overlap of (or similarity among) random sequences groups. In other cases, the random sequences belonging to some communities (i.e. 3, 4, and 5) tend to be more isolated, suggesting a different evolution. Community #1 composed by sequences from lymph nodes form a separate cluster as well as the community #2, composed by tonsils, kidney, pituitary glans and the retropharyngeal lymph node. The largest detected community (#6) is split in two separated branches. The first branch shares the same pattern of the community #2 while the second branch (including only lung and liver tissues) is isolated from the other clusters.

**Figure 5:**
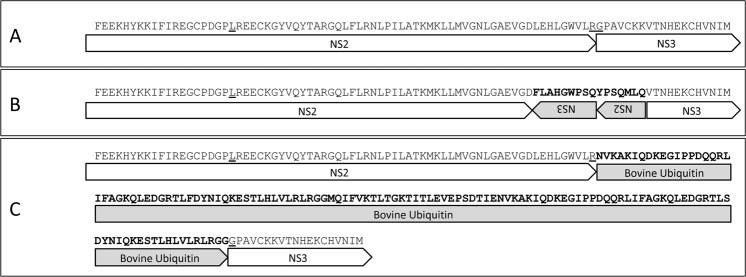
BVDV-2 cytopathic strain characterization. The two variants showing mutated viral sequences are reported in panels B and C, comparing to the non cytopathic strain homologous sequence (panel A). Possible recombination sites are underlined.

During the same outbreak the calf #830 died for MD. Unfortunately only few tissues were available. The NS2-NS3 viral region, described in literature as one of the possible sites involved in the NCP to CP viral mutation^9,35^, was characterized by amplicon sequencing analysis (PCR segment 2 in Table 2). Resequencing procedure was performed using the CN10.2015.821 complete genome as reference. Among the obtained reads it was possible to highlight the presence of the NCP counterpart and at least 2 different heavy mutated variants. The consensus sequence of the NCP strain was highly similar to the homologous region along the #821 full genome. Only 7 synonymous and 2 non-synonymous nucleotide substitutions were recorded, confirming the clonal origin within the herd. In both the mutated strains, a large insertion into the viral genome was present, located around the position B previously reported as possible recombination site^35^. Reads analyses showed the insertion of a 16aa reverted sequence of the NS2-3 viral genome sequence (Fig. 5B) and the insertion of a partial ubiquitin mRNA coding sequence (corresponding to 128 aminoacids, Fig. 5C). In both the cases, the viral open reading frame was respected.

**Table 2:** List of primers used for amplicon sequencing protocol.

Segment	Primers name	Primer 5′-3′ sequence	Position*	Annealing temp	Size
#1	BVDV2 e2_2f	TGGCTGATGCTAATAACAGGGG	2433–2454	56 °C	1569
	BVDV2 NS3_2r	CTTGCTTGAACTCTCCTCTGTTCAT	3984–4006		
#2	BVDV2 p7NS2_2f	GCTGACACACAATGATATTGAGGTTGTGGTC	3620–3650	60 °C	2184
	BVDV2 p7NS2_2r	GTTACATAGCTAGCTAGCAAAAG	5793–5817		
#3	BVDV2 NS5f	AAGGAAGAACCTCAGCAGGGCCACCA	9321–9346	60 °C	1951
	BVDV2 NS5r	CTGTTATCAGAAAGCCATCATCACC	11259–11283		

The positions (*) is based on the Bovine viral diarrhea virus 2 strain CN10.2015.821 sequence [MG879027].

## Discussion

BVDV replication cycle is known to be complex, involving peculiar mechanisms, as the self recognition step as escape strategy against the host immune system. The fetus infected during the pregnancy can become persistently infected as a result of the lack of immune response against the virus. For this reason, it is commonly thought that the evolution of BVDV within the PI host is constrained within the antigenic patterns identified during the self recognition period. This point is clear if we consider the absence of antibodies against BVDV for the entire animal life, but it leads to assume that BVDV immunogenic regions can not mutate, otherwise an immunological response against the evolved viral strains would led to clearance of mutated variants.

The complete genome of strain CN10.2015.821 was obtained after cell culture isolation from serum of the PI cow. The consensus sequence obtained from the three passages were identical. The same result was recorded when the consensus sequences from each tissue were compared. The similarity among sequences obtained from different body comparts is extremely high, suggesting a very strong stability of the infecting viral strain and a very low compartmentalization. The recorded homogeneity among consensus sequences can be partially justified considering that it is not possible to completely remove the serum derived virus from the tissues before RNA extraction, since blood serum is naturally present in all the tissues, as well as the virus carried by it^41^. The only relevant change along the investigated genome regions is the insertion of one amino acid, a lysine, in the E2 protein codified by BVDV in nervous tissues. Considering the role of E2 in the host virus interaction, this result can suggest a different cell tropism needed by the virus in order to infect the nervous system as described for the Hepatitis C Virus (HCV), another member of the Flaviviridae family^41^. On the other hand, the insertion of a new codon along the sequence codifying a structural protein should be considered a substantial evolutionary change, contradicting the idea of genome stability.

If only few differences were highlighted among consensus sequences, a completely different scenario arose when NGS sequencing data analysis was performed. During viral isolation, a large number of variable positions was recorded along the genomes sequences from all cell culture passages. This result suggests a high heterogeneity of the infecting viral strain in the blood serum of PI animal. Moreover, it was possible to identify a high number of different variable sites, characterizing each tissue and proving the existence of different evolutionary processes within them. The largest part of the identified variable positions showed non-synonymous substitutions along the viral polyprotein, in contrast with the previous study^42^ in which Sanger sequencing was performed. This result clearly supports the presence of a viral quasispecies within a BVDV-2 PI animal. Furthermore, this high degree of variability, also at the amino acid level, supports the idea of a viral progeny that can potentially show different antigenic patterns. Analysis of dN/dS ratio revealed that the proportion of amino acids changes is different among the tissues (see Table 1 and Results section for details). The largest part of lymph nodes showed similar results (ω > 1) suggesting potential positive selection, viral adaptation and compartmentalization within the host. On the other hand, the virus tends to remain genetically and antigenically stable in some tissues like the ones belonging to the nervous system (communities #3 to #5) where ω is always lower than one.

Even if protein variation is possible by our results, no antibody response is recorded in PI animals. Forton and colleagues reported a similar framework for HCV. In the study, they concluded that the accumulation of mutations in structural genes can occur in tissues characterized by lower translation levels, since natural selection is exerted by the immune system only after protein translation^41^. BVDV can bypass this biological constrain, using *in utero* infection self recognition as escaping strategy. Moreover, the extremely large number of variants found in our experiments enforces the idea that the congenital infection is carried out by a swarm of variants of the same viral strain which is recognized by the fetus immune system as self as proposed in previous studies^42,43^. This hypothesis supports the fact that the mutation of BVDV is possible even if it must be restricted to the antigenic variations recognized during the pregnancy. In this way, the viral strain infecting a PI animal can move within an evolutionary landscape larger than expected, since the heterogeneity in the viral population can help the compartmentalization and can lead to the tissue adaptation. The compartmentalization is highlighted by the VS analyses: communities in the bipartite network between tissues and VS were detected, and they grouped tissues basing on the genetic variation features. Tissues belonging to the nervous systems shared similar VS distribution as well as lymph nodes, suggesting a different evolution for tissue adaptation as previously reported for HCV virus^41^. On the other hand, the other organs showed a more homogeneous pattern.

These results underline the complexity of BVDV-2 viral population structure within a PI host. The genetic variation found within each tissue indicates the presence of very dynamic viral populations, able to evolve in different directions and suggesting tissue compartmentalization.

Changes occurring along the BVDV viral genome can promote the mutation of a NCP strain in the CP biotype leading to the fatal mucosal disease. Among the mutations that are involved in the NCP to CP biotype transition, several were recorded in the NS2-NS3 viral genome^9,35^ like point mutations or non-homologous RNA recombination leading to the insertion of viral or cellular sequences^44,45^. The analysis of amplicon sequencing data of the NS2-NS3 viral region of the #830 animal dead for the mucosal disease during the same outbreak, showed the presence of both the NCP counterpart and the two heavy mutated variants as previously reported in literature^9,45,46^. Even if it was not possible to isolate di CP strains, the NGS approach allowed to describe at least two mutated and potentially CP BVDV strains within the animal died for MD. Further studies are needed to better understand the mechanisms promoting the NCP to CP biotype mutation and what tissues are mainly involved in this process.

## Materials and Methods

### Sample collection

PI animals were identified as described in a previous work during an outbreak of MD in Cuneo Province, Northern Italy^5^.

In order to genetically characterize the viral strain responsible for the outbreak two different Fresian Holstein calves were included in this study.

The animal #821 was a 6 months old and it was apparently clinically healthy. During regular slaughter process, blood and tissue samples (see Table 1) were collected and immediately frozen at −80 °C.

The second animal (id #830) was a 4 months old calf died for MD. Tissue samples from spleen and duodenal mucosa scraping were collected for the characterization of the NS2-NS3 region of the viral strain responsible for the MD outcome.

All samples collected in the current study were taken in concordance with national legal and ethical regulations applied to regular slaughter procedures, therefore no ethical approval was required.

### Viral isolation and RNA extraction

Blood serum from PI #821 animal was used to infect pestivirus free Madin Darby Bovine Kidney Epithelial (MDBK) cells lines in order to obtain a suitable amount of viral RNA for genome sequencing. Cells were cultured in Dulbecco’s modified essential medium containing 10% of infected serum, 2 mM L-glutamine, 100 IU/ml penicillin, 100 mg/ml streptomiycin and 2.5 mg/ml anphotericin B and 50 ug/ml gentamicin. Cultures were maintained at 37 °C in humidified atmosphere containing 5% of CO_2_ for four passages. Starting from the first passage infected serum was replaced with pestivirus-free fetal bovine serum (FBS, Gibco, Thermo Fisher Scientific). The supernatant of the three further passages was centrifuged at 600 × g for 20 min to eliminate cell debris and was concentrated with Amicon-15 100 kDa centrifugal filter tubes (Sigma Aldrich). Viral RNA was extracted using QIAmp viral RNA mini Kit (Qiagen, Hilden, Germany). Double stranded cDNA was obtained with Maxima H Minus Double-stranded cDNA Synthesis kit (Thermo Fisher Scientific, Whaltam, MA, USA) in accordance with manufacturer instructions and quantified with fluorometric method Qubit dsDNA kit (Life Technologies).

Thirty mg of each tissue sample collected from #821 PI calf were homogenized with TissueRuptor II (Qiagen, Hilden, Germany) and centrifuged; the supernatant was used in order to extract viral RNA using RNAeasy mini Kit (Qiagen) according to the manufacturer’s instructions. Total RNA was reverse-transcribed using Superscript IV Reverse Transcriptase (Thermo-Fisher Scientific) and random hexamers.

The viral RNA was extracted from spleen and gut mucosa of #830 cow with Qiagen RNeasy mini kit (Qiagen, Hilden, Germany). The single stranded cDNA was synthetized using Invitrogen Superscript IV Reverse Transcriptase (Thermo Fischer Scientific) and used as template for the amplification of the viral genomic region including NS2-3 genes.

### Primer design and PCR amplification

Six oligonucleotide primers (Table 2) were used to amplify three regions of the viral genome, including E2, NS2, NS3 and NS5 genes, using Invitrogen Platinum Taq Polymerase High Fidelity (Thermo Fisher Scientific) in a 50 µl reaction, starting from the synthetized cDNA.

After the initial denaturation step of 1 minute at 94 °C the amplification profiles included 30 cycles of 30 seconds at 94 °C, 30 seconds at the annealing temperature (see Table 2) and the elongation step at 68 °C (see Table 2). The final elongation step was 10 minutes long at 68 °C.

Eight microliters of PCR product were run on 1.5% agarose gel stained with Invitrogen SyberSafe reagent (Thermo Fisher Scientific) to confirm the presence of the band of expected size (Table 2). Fourty microliters of PCR product were purified with Nucleospin Gel and PCR clean up kit (Macherey-Nagel, Germany), eluted in 20 µl of RNAse-free water and quantified with fluorimetric method Qubit High Sentitive dsDNA kit (Life Technologies).

### MiSeq run

Pooled equimolar amplicons as well as the double stranded cDNA obtained from the MDBK cell culture were sequenced on MiSeq platform in a V2 500-cycles kit.

All samples were prepared for NGS using Nextera XT DNA Library Preparation Kit (Illumina, San Diego, CA, USA), according to the manufacturer protocol. The library concentration and quality was evaluated with fluorimetric method Qubit High Sentitive dsDNA kit (Life Technologies) and with Agilent DNA High Sensitivity chip assay.

### MiSeq reads analysis

Raw sequencing reads were first filtered for quality (FastQC) and trimmed (Trimmomatic). The remaining FastQ reads were then assembled into contigs using Velvet Genome Assembler. Assembled contigs were compared for similarity against all viral sequences available in the NCBI GenBank nucleotide database using BLASTn. The results were visualized using Geneious software (vers 11.1.2). Raw reads were also aligned to the available reference BVDV-2 genomes using Geneious software. A consensus sequence was then obtained and confirmed through a second resequencing step. Variant sites and their frequency were recorded during this step, in order to evaluate viral heterogeneity during the viral isolation in cell culture.

The consensus sequence of the complete viral genome obtained from the viral isolation was submitted to GenBank with the accession number MG879027^47^. The complete genome sequence, representing the NCP biotype, was used as reference during the analyses of reads obtained from #821 (all tissues in Table 1) and #830 (spleen and duodenal mucosa scraping) amplicon sequencing experiments, by using mapper algorithm embedded in Geneious software. The first resequencing step, used for the consensus generation, was performed with low sensitivity/fastest and none fine tuning (no iterations) parameters.

Once the consensus sequence was obtained for each tissue the reads were newly aligned with low sensitivity/fastest and fine tuning (5 iterations). Variants along the alignment were identified if their coverage was larger than 100x and the frequency was higher than 1%. The characterization of CP biotype in #830 samples was carried out isolating the aligned reads that showed part of their sequence largely different from the consensus. In more details, alignment algorithm sensitivity allowed to identify those reads belonging to CP variants, because part of the read was highly similar or identical to the homologous region in the NCP strain.

### Sequence analyses

The #821 complete genome sequence was aligned with a set of available reference sequences, including both BVDV-1 and BVDV-2 strains. Phylogenetic relationships among sequences were inferred using Bayesian approaches (MrBayes software^48^), based on the best evolutionary model (jModelTest^49^).

Nucleotide diversity among tissues consensus sequences was evaluated as uncorrected p distance. Quasispecies heterogeneity was investigated considering each variable site within the amplified regions. Each tissue was characterized by the position, the frequency, and the type of mutation (synonymous or non synonymous mutation and involved nucleotides) of each VS. The number of non synonymous substitutions per non synonymous site (dN), as well as the number of synonymous substitutions per synonymous site (dS) and their ratio (ω) were calculated using SNPGenie software^50^; values for each tissue are reported in Table 1.

Patterns of VS were used to compare tissues by cluster analysis: distance matrix based on the presence/absence of VSs was calculated using Dice similarity index. Complete linkage method was used to draw the cladogram describing the similarity among tissues based on VS distribution (MASS package in R^51^). The same approach based on shared VS positions was used to draw a bipartite network, in order to evaluate a possible structure in the viral population. The network was built considering tissues and VS as two separated families of nodes and edges were weighted on the VS frequency and type. With this approach two tissues are connected only if they share at least one VS. Network was built using *ade4* and *igraph* packages in R^51^, with hand written pipelines. Communities detection was conducted with the Beckett algorithm implemented in the *bipartite* package in R^51^.

For further describe viral diversity a different experimental approach was used. A set of random sequences was created for each tissue. A total of 100 sequences were generated as follow for each tissue: starting from the tissue consensus sequence one VS was randomly selected and changed basing on the substitution frequency recorded during amplicon sequencing analyses. In this way, the set of 100 random sequences included variants deriving from the quasispecies heterogeneity. The random sequences belonging to all the tissues (n = 2500) were aligned and uncorrected p distance matrix was generated. Multidimensional scaling was generated in order to describe the position of the group of sequences belonging to each tissue (R *mass* package).
